# In the Arms of Morpheus: Recent Advances in Dreaming and in Other Sleep-Related Metacognitions

**DOI:** 10.3390/brainsci14101017

**Published:** 2024-10-14

**Authors:** Sérgio Mota-Rolim, Brigitte Holzinger, Michael R. Nadorff, Luigi De Gennaro

**Affiliations:** 1 Brain Institute, Federal University of Rio Grande do Norte, Natal 59078-970, Brazil; 2 Institute for Consciousness and Dream Research, 1180 Vienna, Austria; 3 Department of Psychology, Mississippi State University, Starkville, MS 39762, USA; 4 Department of Psychology, Sapienza University of Roma, 00185 Roma, Italy

Dreams have always fascinated humans. The first written references to dreaming come from Greek mythology: Nyx, the goddess of night, gave birth to Hypnos, who represented sleep. Hypnos had a twin brother called Thanatos, the god of death, and fathered Morpheus, the god of dreams. In addition, most religions, such as the Abrahamic monotheisms—Judaism, Christianity, and Islam—recognize dreams as a way to communicate with God to understand the present and predict the future. Thus, ancient civilizations tend to interpret dreams as supernatural phenomena [1].

This began to change with Freud. In 1900, his famous book “*The Interpretation of Dreams*” was the first attempt to understand dreams as a natural process of the brain [2]. Nowadays, dreams are currently defined as any mental activity that occurs during sleep. More specifically, dreams are sense–perceptual, emotional, and cognitive experiences that are more frequently recalled after awakening from rapid eye movement (REM) sleep [3,4,5,6,7]. However, dreams can also occur during non-REM (NREM) sleep stages, with specific characteristics. During sleep onset (N1), dreams tend to be fast, and appear as unspecific images and sounds, or strange ideas. Dreams during light sleep (N2) are more related to the thoughts and memories of the previous day(s). During deep sleep (N3), dreams are remembered less often [8,9,10,11,12,13,14].

Despite all the significant scientific advances in dream research in the last century, we still do not know why we dream, and why some people remember dreams every day, while others only rarely. Modern theories such as the continuity hypothesis [15], the threat-simulation theory [16], the activation hypothesis [17,18], and neuropsychoanalysis [5] further our understanding of dreaming, but they cannot explain all oneiric features.

For this Special Issue, we invited experts in dream research to submit original research articles and review papers. We aimed to provoke and articulate ideas to foster a broad discussion on dream research, and to convey the challenges and misunderstandings in this complex research field. In addition to stimulating the submission of neuroscientific studies, we also encouraged philosophical works, because we believe that this interaction between biomedical and social sciences is fundamental to fostering our understanding of dreams and human consciousness. In all, ten papers—including six original research articles and four review works—were published in this Special Issue.

As previously stated, typical dreams are more related to REM sleep. However, according to the comprehensive review of Pagel (contribution 1), there are still many controversies about this sleep state. REM dreams are longer, more bizarre, transformative, and remembered, but none of these characteristics are exclusive to REM sleep. Moreover, REM sleep is also known as paradoxical sleep, because it presents the intracranial electrical theta rhythm (5-8Hz), but this oscillation is the marker of REM sleep in non-human animals. Thus, REM sleep and dreaming continue as a paradox, and more work directly addressing the electrophysiology of REM sleep and the phenomenological features of REM dreams is necessary.

Dreams include perceptual experiences, but what are the main sensory modalities that appear in dreams? To clarify this issue, van der Heijden et al. (contribution 2) developed a dream diary with questions about sensory experiences during dreaming, and found that visual modality was the most common, followed by auditory and tactile ones. Olfactory and gustatory modalities had similar lowest rates. These results, at least in terms of sensorial and perceptual modalities, are in accordance with the continuity hypothesis [15], which postulates that dreaming is similar to the waking life.

Dreams are also relevant for clinical issues. In historical terms, the German philosophers Kant and Schopenhauer suggested that “a lunatic is a wakeful dreamer” and that “a dream is a short-lasting psychosis, and a psychosis is a long-lasting dream”, respectively. Wundt, who was also one of the founders of experimental psychology, affirmed that “we can experience in dreams all the phenomena we find in the hospice”. All of these authors influenced Freud, who postulated that psychosis is an abnormal intrusion of a dreaming activity into waking life. In this way, dreams can be seen as a model of psychosis, since both include internally generated perceptions and lack of rational judgment, probably due to the decreased activity of the frontal regions during REM sleep [19]. Ficca et al. (contribution 3) assessed the dream reports of subjects diagnosed with schizophrenia using both quantitative and qualitative methods and found correlations with illness severity.

Kempe et al. (contribution 4) observed, preliminarily, that the improvement in personality functioning during psychotherapy is associated with an increased ability to regulate the affects one experiences during dreams. Additionally, Desjardins et al. (contribution 5) investigated the relation between dreams and morning mood in adolescents hospitalized after a suicide attempt. Consistent with the previous literature [20], the authors found that suicidal adolescents had nightmares more frequently, and that their dreams had more negative moods, destructive themes, failures, and aggressions.

In the last few years, an increase in dreams’ and nightmares’ recall frequency was also observed due to the COVID-19 outbreak [21,22,23]. Camaioni et al. (contribution 6) investigated how the COVID-19 pandemic affected sleep talking and dreams, and found a higher frequency of sleep talking episodes during the pandemic compared to before the pandemic. In addition, the authors also observed that sleep talking episodes were associated with the emotional intensity of dreams, independent of the pandemic.

An interesting kind of dreaming that has been gaining more attention recently is lucid dreaming, when subjects know that they are dreaming during the dream, and may also have some degree of control over the oneiric plot [24]. However, the main challenge in lucid dream research is developing a reliable way to induce them, because they are rare for most people. Since lucid dreaming has similarities with the notion of mindfulness and meta-awareness, Gerhardt and Baird (contribution 7) investigated its association with meditation, and found that daily frequent meditators experience more lucid dreams than non-frequent meditators, and that meta-awareness is higher for meditators and weekly lucid dreamers. In addition to practicing meditation, another way to induce lucid dreams is trough exogenous substances. Oldoni et al. (contribution 8) reviewed natural plants and artificial drugs that increase metacognition, REM sleep, and/or dream recall. The authors found that the main candidates are substances that increase cholinergic and/or dopaminergic transmission, such as galantamine.

We also received an important philosophical work by Kuiken (contribution 9), who reviewed the epistemic significance of impactful dreams, dividing them in nightmares, existential dreams, and transcendent dreams. The author also defines a neo-Kantian account of “sublime feeling” as the cumulative effect of successive metaphoric/literal categorical transformations that produces a higher-level form of metacognition. Sublime feeling occurs as either sublime disquietude (existential dreams) or as sublime enthrallment (transcendent dreams).

Finally, one of the main features of contemporary urban societies is that everybody has a smartphone. In a scoping review, Diushekeeva et al. (contribution 10) investigated the impact of pre-sleep visual media exposure on dreams, and found that the range of stimulus-related incorporation was 3–43% for REM dream reports, and 4–30% for NREM dream reports. In addition to entering dreams, smartphone use is forcing people to sleep later, but subjects still need to go to school, university, or work early in the morning. Since REM sleep episodes tend to be longer in the second half of the night, modern humans are partly deprived of this sleep stage [25]. Recent works have shown that REM sleep and dreaming are associated with emotional regulation [26]; thus, we hypothesize that the contemporary increase in depression diagnostics may be related to this REM sleep deprivation. We also hypothesize that this emotional regulation failure caused by dream deprivation may also contribute to our insensibility in destroying the natural resources that constitute our planet, and that are fundamental for all living beings, including us humans. Future works are necessary to clarify this crucial issue.
